# Adopting a couples’ approach to treating chronic cancer-related fatigue: a single-arm pilot trial of a web-based cognitive therapy for couples

**DOI:** 10.1007/s00520-026-10547-8

**Published:** 2026-03-16

**Authors:** Mariët Hagedoorn, Fabiola Müller, Sophie I. van Dongen, Rosalie A. M. van Woezik, Marrit A. Tuinman, Melanie P. J. Schellekens, Marije L. van der Lee

**Affiliations:** 1 Department of Health Sciences, Section Health Psychology, University of Groningen, University Medical Center Groningen, Hanzeplein 1 (HPC FA12), 9700 RB Groningen, The Netherlands; 2 Department of Public & Occupational Health, Amsterdam, UMC Location University of Amsterdam, Meibergdreef 9, 1105 AZ Amsterdam, the Netherlands; 3 Cancer Treatment and Quality of Life, Cancer Center Amsterdam, Amsterdam, the Netherlands; 4 Department of Scientific Research, Helen Dowling Institute (Center for Psycho-Oncology), Bilthoven, The Netherlands; 5 Department of Medical and Clinical Psychology, Tilburg University, Tilburg, The Netherlands

**Keywords:** Couples therapy, Mindfulness, Psycho-oncology, Cancer, Chronic fatigue, Single-arm pilot

## Abstract

**Purpose:**

Many cancer survivors experience chronic cancer-related fatigue (CCRF). While most psychosocial interventions focus exclusively on cancer survivors themselves, increasing evidence suggests that involving partners may enhance and broaden the benefits of these interventions. The primary objective of this study was to assess the acceptability and potential efficacy of COMPANION, a web-based mindfulness-based cognitive therapy for couples (15–20 weeks). Additionally, we examined the feasibility of the trial procedures.

**Methods:**

This single-arm pilot trial included cancer survivors and their romantic partners. Participants completed questionnaires prior to starting the therapy (T0), 2 weeks after the therapy (T1), and 1 month following T1 (T2). Predefined benchmark values were used to assess the acceptability and potential efficacy of COMPANION, as well as the feasibility of the trial procedures.

**Results:**

Forty-six couples were screened for eligibility, 33 fulfilled the inclusion criteria and 21 entered the study. Seventy-one percent of the couples completed COMPANION (*n* = 15). Most couples were satisfied with the intervention and the couples’ approach. About two-thirds of the cancer survivors showed a clinically relevant improvement in fatigue from T0 to T1. Improvement was also seen in anxiety and depressive symptoms in both members of the couples. The trial procedures were found to be feasible.

**Conclusions:**

Based on the benchmark values, we conclude that the intervention is acceptable and potentially efficacious. A randomized controlled trial is therefore warranted to further evaluate the effectiveness of COMPANION.

**Implications for cancer survivors:**

After further testing, the intervention may be offered more broadly and complement much-needed psychosocial interventions to help couples cope with CCRF.

**Supplementary Information:**

The online version contains supplementary material available at 10.1007/s00520-026-10547-8.

Chronic cancer-related fatigue (CCRF) is a prevalent and distressing symptom experienced by cancer survivors, which is characterized by physical, emotional, and/or cognitive tiredness or exhaustion that is disproportionate to recent activity levels [1–3]. Approximately one in four cancer survivors continues to experience this fatigue for months or even years following the completion of cancer treatment [3]. CCRF frequently disrupts normal functioning, making it more challenging for cancer survivors to work, perform everyday tasks, and sustain social relationships [4, 5]. Not surprisingly, this persistent fatigue is associated with reduced quality of life and increased distress [3, 6]. The underlying mechanisms of CCRF are likely multifactorial and complex, involving a combination of molecular/physiological, clinical, and psychosocial factors [3, 7, 8]. Non-pharmacological treatments have been found to be more effective than pharmacological treatments, with especially psychological and exercise therapies being at least modestly successful in relieving fatigue [3, 9, 10]. Notably, these therapies have traditionally focused exclusively on cancer survivors [11], while emerging evidence points to the importance of involving romantic partners in CCRF management [12–14].

More specifically, cancer survivors cope with cancer and CCRF within the context of their close relationships. Interactions with loved ones, especially intimate partners, may significantly influence fatigue outcomes [12–14]. For instance, increased fatigue has been reported on days when cancer survivors engage in ruminative discussions with their partners [12], whereas days characterized by partners’ facilitating behaviors—such as encouragement to be active—and fewer solicitous behaviors—such as taking over chores—have been linked to reduced interference of fatigue in daily life [13]. Moreover, the benefits that cancer survivors receive from psychological therapy for fatigue may depend on factors related to their partner and relationship. For example, higher partner fatigue and greater patient relationship dissatisfaction have been associated with poorer treatment outcomes in cognitive-behavioral interventions for chronic fatigue [14]. These observations suggest that involving the partner in therapy and fostering supportive daily dynamics between cancer survivors and their partners could optimize fatigue management for cancer survivors. Furthermore, as the cancer experience and CCRF can also adversely affect intimate partners [4, 15, 16], learning to manage fatigue together may benefit both partners’ well-being and potentially enhance the quality of their relationship.

Although we have seen growing attention for interventions for couples, most couples interventions focus broadly on cancer-related or unmet needs, rather than specifically targeting CCRF [17–20]. The present study addresses this gap by evaluating a couples-based intervention—COMPANION—that specifically targets CCRF. COMPANION was developed by adapting a previously validated, 9-week, web-based, therapist-guided individual mindfulness-based cognitive therapy (eMBCT), which has shown efficacy in reducing CCRF [21]. Recent meta-analytic evidence shows that mindfulness-based cognitive therapy surpasses cognitive-behavioral therapy and psychoeducation in effectiveness for CCRF [22]. The adaptation of the individual eMBCT into COMPANION was informed by a mixed-methods study examining couples’ preferences and wishes regarding such a joint therapy [23].

The primary goal of the current study was to examine the acceptability of COMPANION and its potential efficacy in reducing fatigue in cancer survivors. Secondary objectives included evaluating the feasibility of the trial procedures and exploring the potential efficacy of COMPANION in promoting emotional and relational well-being in cancer survivors as well as their partners.

## Methods

### Study design

This paper reports on a single-arm pilot study involving cancer survivors with CCRF and their romantic partners. All participants received COMPANION, a web-based mindfulness-based cognitive therapy for couples. Both cancer survivors and their partners completed questionnaires prior to the start of the therapy (T0), 2 weeks after completing the therapy (T1), and 1 month following the initial follow-up (T2). The trial was pre-registered at ClinicalTrials.gov (NCT05636696) and details can be found in the protocol paper [24].

### Participants

Couples were eligible to participate if one member had received a cancer diagnosis, had finished cancer treatment at least 3 months ago (excluding ongoing hormone therapy), and had been experiencing severe fatigue for a minimum of 3 months (self-reported). A complete overview of inclusion and exclusion criteria can be found in the study protocol [24].

### Recruitment

Participants were recruited at the Helen Dowling Institute (HDI), the University Medical Center Groningen (UMCG), and through self-referral. The research assistant contacted cancer survivors who were waiting for patient-centered eMBCT at the HDI and who had provided consent to be approached for research. Cancer survivors at the UMCG were informed about the study by their oncologist or nurse. Subsequently, those who showed interest were contacted by the research assistant. Recruitment through self-referral was promoted through several channels, such as messages on social media, articles in newspapers, and an invitation sent to the Kanker.nl panel [25].

### Screening

The research assistant provided additional information about the study to all interested couples and screened their eligibility during an online interview. If both the cancer survivor and their partner provided informed consent, the cancer survivor was sent an email with a link to a survey to confirm whether the eligibility criteria were met.

### Assessments

All questionnaires were administered via REDCap (Research Electronic Data Capture), a secure, web-based application [26]. Both cancer survivors and their partners received survey links by email and were encouraged to complete the questionnaires independently. Reminder emails were sent if a participant did not respond; the first after 7 days, followed by a second reminder after an additional 7 days if necessary. Therapists maintained a log to document intervention delivery, while the research assistant kept a separate log to track recruitment and study adherence.

### Sample size calculation

The study was designed to evaluate the potential efficacy of COMPANION in reducing fatigue in cancer survivors, which was the second primary research objective. Changes in fatigue severity among cancer survivors from baseline (T0) to the first (T1) follow-up were analyzed using a one-tailed paired *t*-test on scores from the Fatigue Severity subscale of the Checklist Individual Strength (CIS-fatigue). To optimize statistical power—and because existing evidence suggests an increase in average fatigue is unlikely—a one-tailed test was used [21]. G*Power analysis indicated that a sample size of 27 couples would be needed to detect a medium effect (0.5) with an alpha level of 0.05 and a power of 0.8 (one-tailed test).

### Intervention

COMPANION is a web-based, therapist-guided couples therapy designed to help cancer survivors modify their cognitive and behavioral responses to cancer-related fatigue. The therapy incorporates psychoeducation as well as mindfulness and cognitive-behavioral exercises. It is based on the MBCT protocol by Segal, Williams, and Teasdale [27] and adapted from the eMBCT developed by Bruggeman-Everts et al. [21]. The unique aspect of COMPANION is its couples-based approach, as it actively involves the partner in the therapeutic process. Specifically, the therapy includes (1) three video call sessions involving the cancer survivor, their partner, and the therapist; (2) psychoeducation for the partner; (3) additional exercises for both the cancer survivor and partner to complete together or individually; and (4) an extra session dedicated to the couple’s relationship and mindful communication about fatigue. In collaboration with the therapist, couples can determine the manner and extent of the partner’s involvement. Cancer survivors use a secure web-based platform to access written information related to the central theme of each session (e.g., fatigue and the automatic pilot, dealing with boundaries, communication). Partners can view these materials via the cancer survivor’s account. Further details about the themes of the sessions are provided in the study protocol. There are nine sessions to be completed over 15 to 20 weeks, with flexible scheduling to accommodate holidays and other commitments.

### Therapist training and treatment integrity

COMPANION was delivered by trained licensed therapists at the HDI. Therapist training included a self-study module to become familiar with the web portal, as well as interactive training sessions focused on the therapy content and the therapist’s role within the therapy. Therapists also received instruction on the assessment procedures used in the COMPANION trial, such as how to complete the therapist log. All procedures are detailed in a therapist manual, and ongoing supervision was provided throughout the trial.

### Measures

#### Demographics and cancer-related characteristics

Demographic variables and cancer survivors’ cancer-related variables were self-reported at the screening and/or T0.

#### Acceptability of COMPANION

*Intervention adherence* was evaluated at T1, based on self-reports from cancer survivors and data recorded by therapists in their logs. Reasons for discontinuing the intervention could be noted in an open-text field. Completion of the intervention was defined as the cancer survivor attending at least six out of nine sessions, with the partner not having dropped out (as confirmed by both self-report and the therapist log). The therapist assessed the partner’s level of involvement on a 7-point Likert scale, with scores of 5 to 7 indicating (high) involvement. Additionally, both the partner and the therapist provided information about the ways in which the partner participated in the therapy.

*Intervention satisfaction* was measured at T1 using two items: overall satisfaction with the COMPANION therapy (“Overall, how satisfied are you with the COMPANION therapy?”) and satisfaction with the couples’ approach (“How satisfied are you with the possibility of jointly participating in the COMPANION therapy?”). Additional items assessed satisfaction with specific elements of the intervention and the partner’s involvement. All items were rated on a 7-point Likert scale, with scores of 5 to 7 indicating (high) satisfaction. Negatively worded items were reverse-scored.

#### Fatigue severity in cancer survivors

*Fatigue severity* during the past 2 weeks was assessed using the eight items of the Fatigue Severity subscale of the Checklist Individual Strength (CIS-fatigue scale; e.g., “I feel tired”), each rated on a 7-point Likert scale from (1) “Yes, that is true” to (7) “No, that is not true.” These assessments were administered at all measurement points. Higher total scores (Cronbach’s *α* = 0.835) reflect more severe fatigue, with possible scores ranging from 8 to 56. A score of 35 or higher indicates severe fatigue in cancer survivors [28, 29]. A clinically relevant change was defined as a difference of 6 points or more [30].

#### Feasibility of trial procedures

To evaluate the *feasibility of recruitment*, the research assistant maintained a recruitment log documenting (1) the number of participants (i.e., cancer survivors and/or partners) who expressed interest in participating (i.e., the number of couples screened for eligibility); (2) the number of couples found eligible for the study; (3) the number of couples actually enrolled in the study; (4) the number of couples who discontinued the intervention or withdrew from the study; and (5) the reasons for ineligibility and decline of participation. The research assistant documented *adherence to the study protocol* in the recruitment log, recording both the number and the percentage of questionnaires completed. We defined completion of the questionnaires as having provided responses to at least the fatigue questionnaire and the two primary satisfaction items (i.e., the outcome measures).

#### Well-being in cancer survivors and partners

*Anxiety* and *depressive symptoms* were assessed in both cancer survivors and partners using the Hospital Anxiety and Depression Scale (HADS) [31, 32]. The 14 items were answered on a Likert scale ranging from 0 to 3. Example items include “I feel tense or wound up” (anxiety) and “I feel as if I’m slowed down” (depressive symptoms). Higher total scores indicate more anxiety (Cronbach’s *α* = 0.847) and depressive symptoms (Cronbach’s *α* = 0.853). To assess *relationship satisfaction*, both cancer survivors and partners completed the marital quality subscale of the Maudsley Marital Questionnaire [33, 34], which consists of ten items rated on a 9-point Likert scale (0 to 8). An example item is “Do you get enough warmth and understanding from your partner?” A higher total score indicates greater relationship satisfaction (Cronbach’s *α* = 0.847). All assessments were administered at each assessment point. 

### Analyses and benchmark and critical values

To determine whether proceeding to a randomized controlled trial (RCT) is warranted, we established predefined benchmarks and critical values. Benchmarks indicate the minimum desirable outcomes, while critical values — set at half the benchmark values — highlight potential concerns with the outcome assessed. These thresholds were set in advance, informed by existing literature and our clinical and research experience (see the study protocol for further details) [24].

#### Acceptability of COMPANION

The benchmark value for intervention adherence was at least 60% of couples completing the intervention, while the critical threshold was set at less than 30% completion. These values were informed by the previous patient-centered trial that reported 38% of intervention dropout [21] as well as comparable web-based couple interventions [35, 36].

For satisfaction, the benchmark was that at least 70% of the cancer survivors and partners reported being satisfied with the intervention overall and/or the couples’ approach. The critical value was set at less than 35% of the cancer survivors and partners being satisfied. Analyses included all couples who started the therapy, regardless of completion of the intervention. These values were based on a previous couple intervention study [20], where acceptability of different intervention components ranged between 75 and 100% for cancer survivors and family members.

#### Potential efficacy in reducing fatigue in cancer survivors

Support for the potential efficacy of COMPANION was defined as a statistically significant reduction (paired *t*-test) and a clinically relevant improvement (a decrease of at least 6 points on CIS-fatigue) in cancer survivor fatigue from baseline (T0) to first (T1) follow-up. Analyses were conducted on both an intention-to-treat (ITT, including all couples allocated) as well as per-protocol (PP, all couples who completed the therapy) basis. Missing fatigue severity data at T0, T1, and T2 were imputed, based on demographics and baseline clinical characteristics. Five imputations were performed using fully conditional specification (MCMC) with up to ten iterations, and the resulting datasets were pooled for analysis. Additionally, sensitivity analyses were conducted on participants with non-missing CIS-fatigue T0 and CIS-fatigue T1 values. A clinically relevant improvement was defined as at least 45% of the cancer survivors showing improvement (ITT), while the critical threshold was set at less than 23% improvement. These benchmark values were derived from our previous trial, where 49% of patients assigned to patient-centered eMBCT experienced reduced fatigue (ITT) [21]. Changes in fatigue from baseline (T0) to second (T2) follow-up were explored.

#### Feasibility of the trial procedures

We present a study flowchart to show the number of couples that were screened, found eligible, enrolled, and retained in the study, along with the reasons for non-eligibility and dropout. We also calculated the percentage of cancer survivors and partners who completed and returned the T1 and T2 questionnaires. The benchmark values for questionnaire adherence were set at ≥ 65% completion of questionnaires at T1 (at least fatigue, satisfaction overall and satisfaction with the couples’ approach) and ≥ 60% for T2. Critical values were defined as < 33% completion for T1 and < 30% for T2. For comparison, in our previous trial of the individual eMBCT, 73% of cancer survivors completed both T1 and T2 questionnaires [21]. Although the follow-up period in the current trial is shorter than in similar trials (i.e., 4 weeks), the benchmark for completion of the questionnaires was set conservatively, considering the intensity of the weekly assessments (i.e., up to 22 diaries; see protocol deviations below).

#### Potential efficacy in improving well-being in cancer survivors and partners

The potential efficacy of COMPANION in enhancing well-being among cancer survivors and their partners was explored by examining statistically significant reductions in anxiety and depressive symptoms, as well as statistically significant increases in relationship satisfaction from baseline (T0) to the first (T1) and second (T2) follow-up. These analyses were performed on unimputed data of participants who completed these questionnaires. 

#### Deviations from the study protocol

Due to the limited recruitment period (January–March 2024), we were unable to reliably estimate the recruitment rate at the participating hospital as originally planned. The study incorporated weekly assessments to explore potential mechanisms of change over time, such as changes in mindfulness and partner interactions. Our initial plan was to analyze whether changes in these potential mechanisms co-occurred with changes in fatigue. However, the sample size was too small to support such complex analyses; and therefore, these results are not reported here. Additionally, although we initially planned to conduct a focus group with therapists and interviews with couples, this was not feasible due to time and resource constraints. Importantly, therapist logs and satisfaction questionnaires already yielded sufficiently detailed information to address our research questions regarding the experiences of both therapists and participating couples.

## Results

### Descriptives

**Fig. 1:**
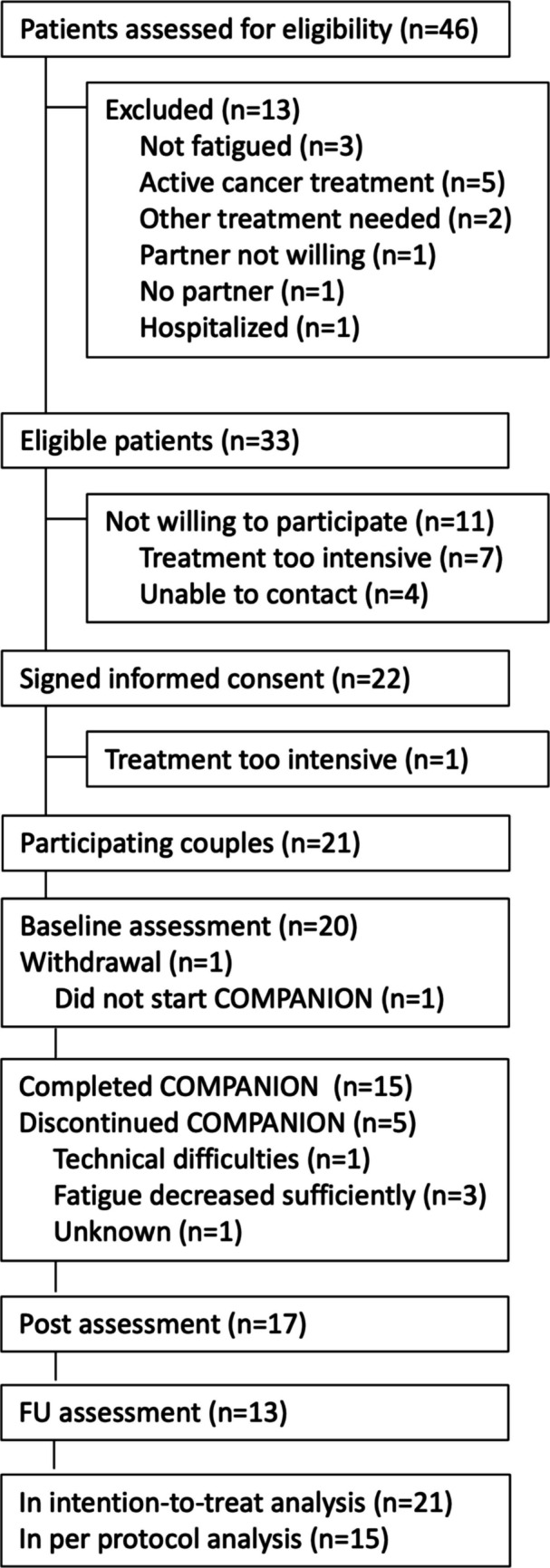
Flowchart of the study

The flowchart (Fig. 1) presents the recruitment process, including reasons for non-eligibility and therapy dropout. Forty-six couples were screened for eligibility, of whom 33 (72%) met the inclusion criteria and 21 (64%) enrolled in the study. Most couples consisted of a female cancer survivor with a male partner (*N* = 16) in their mid-50s (mean age of 54 and 56 years for cancer survivors and partners, respectively). Table 1 presents more details on the characteristics of the couples who completed the baseline questionnaire (T0).

**Table 1:** Sample characteristics of the participants in COMPANION at baseline (*N* = 20)

**Characteristics**		**Cancer survivors** ^a^	**Partners** ^a^
Age, in years, *M (SD), range*		54.3 (10.5) 34–74	56.1 (9.9) 38–76
Sex, female, *N (%)*		16 (80.0)	3 (15.8)
Married, *N (%)*		15 (75.0)	
Relationship duration, *N (%)*			
	5 to 10 years	1 (5.0)	
	10 years and more	19 (95.0)	
Level of education, *N (%)*			
	Middle vocational	4 (20.0)	7 (36.8)
	Higher vocational	7 (35.0)	5 (26.3)
	University	8 (40.0)	7 (36.8)
	Other	1 (5.0)	-
Occupation, *N (%)*			
	Working	8 (40.0)	14 (73.7)
	Retired	3 (15.0)	4 (21.1)
	Unable to work	6 (30.0)	-
	Other	3 (15.0)	1 (5.3)
Cancer type^b^, *N (%)*			
	Breast	8 (40.0)	
	Colon	2 (10.0)	
	Leukemia	1 (5.0)	
	Cervix	1 (5.0)	
	Skin	1 (5.0)	
	Pancreas	1 (5.0)	
	Other	7 (35.0)	
Time since diagnosis, *N (%)*			
	Less than 1 year	2 (10.0)	
	1 to 2 years	6 (30.0)	
	2 to 5 years	7 (35.0)	
	5 years and more	5 (25.0)	
Treatment received, *N (%)*			
	Surgery	17 (85.0)	
	Chemotherapy	14 (70.0)	
	Radiotherapy	11 (55.0)	
	Immuno	4 (20.0)	
	Hormonal therapy completed/ongoing	2/5 (10.0/25.0)	
Treatment completed, *N (%)*			
	Less than 3 months	1 (5.0)	
	3 to 6 months	4 (20.0)	
	6 to 12 months	2 (10.0)	
	More than 12 months	14 (65.0)	
Prognosis, *N (%)*			
	Cancer is/can be cured	17 (85.0)	
	Curation not possible	2 (10.0)	
	I do not know	1 (5.0)	
Metastases, *N (%)*			
	Yes	6 (31.6)	
	No	11 (57.9)	
	I do not know	2 (10.5)	
Comorbidities, *N (%)*			
	Yes	10 (50.0)	14 (70.0)
	No	10 (50.0)	6 (30.0)
Fatigue onset, *N (%)*			
	Before the cancer diagnosis	3 (15.0)	
	Around the time of diagnosis	1 (5.0)	
	During cancer treatment	12 (60.0)	
	After cancer treatment	2 (10.0)	
	Other	2 (10.0)	
Fatigue duration, *N (%)*			
	3 to 6 months	1 (5.0)	
	6 months to 1 year	4 (20.0)	
	1 to 2 years	3 (15.0)	
	More than 2 years	12 (60.0)	

^a^Unless indicated differently. For one partner, baseline data on demographic variables is missing

^b^The numbers do not add up to the total sample size and the percentages do not add up to 100%, as some participants indicated multiple cancer diagnoses

### Acceptability of COMPANION

Seventy-one percent of the participating couples completed the intervention (*N* = 15 of 21), which exceeds the benchmark value of 60%. One couple did not start with the therapy. Three out of the five couples who started the therapy but did not complete it stopped because they felt that the fatigue had decreased sufficiently (see Fig. 1). The therapists evaluated the degree of involvement of the partner in the therapy to be high (i.e., a score of ≥ 5 out of 7) in 75% of the couples (Mean = 5.3, range = 2–7). Involvement consisted mostly of reading information from the therapy, discussing the information with the cancer survivor, performing exercises together with the cancer survivor or individually, and having contact with the therapist (see Appendix A, Table 1, for more details).

The majority of the cancer survivors who started with the therapy were satisfied. Specifically, of the 20 cancer survivors, 14 (70.0%) and 13 (65.0%) scored a 5 or higher on overall satisfaction with the therapy and satisfaction with the couples’ approach, respectively. These percentages are around the benchmark value of 70%. For partners, these percentages are 40.0%, which is below the benchmark value but above the critical value of 35%. Noteworthy, almost half of the partners (i.e., 9 out of 20) did not complete the satisfaction questions. Of those who did complete the T1 questionnaire, more than 70% reported being satisfied (see Table 2 for more details).

**Table 2:** Intervention satisfaction

Outcome measures at T1		Overall satisfaction		Satisfaction with the couples’ approach	
		Cancer survivors	Partners	Cancer survivors	Partners
Mean^a^ (SD) Satisfaction (*N* = 17 CS, 11 P)		5.4 (1.5)	5.4 (1.4)	5.2 (1.7)	5.8 (1.5)
Number satisfied		14	8	13	8
% Satisfied					
	Completed T1 (*N* = 17 CS, 11 P)	82.4	72.7	76.5	72.7
	Started therapy (*N* = 20)	70.0	40.0	65.0	40.0
	Entered the study (*N* = 21)	66.7	38.1	61.9	38.1

*CS*, cancer survivors; *P*, partners

^a^The scores range from 1 to 7, with a higher score indicating a higher level of satisfaction. Scores of 5 to 7 indicate (high) satisfaction

At least half of the cancer survivors and partners who completed the questionnaires indicated that they felt that the therapy has helped more because the partner was involved (47% and 64%, respectively), liked that they could decide themselves to which degree the partner was involved (59% and 82%, respectively), felt that the partner now better understood what it is like to be fatigued (65% and 64%, respectively), felt that the partner had encouraged the cancer survivor to start or continue the treatment (53% and 45%, respectively), perceived that doing the treatment together had a positive effect on their relationship (65% and 55%, respectively), and would recommend COMPANION to other couples (76% and 45%, respectively). The treatment helped the cancer survivors to cope better with the fatigue (71% and 55%, respectively), while the partners also received attention for problems they were facing (65% and 64%, respectively). Although they found the time investment acceptable (65% for cancer survivors and 64% for partners), the most frequently mentioned challenge was finding the time for participating in the treatment together (29% of the survivors and 18% of the partners were satisfied with this aspect). Appendix B (Table 2) presents more details on satisfaction with different aspects of the therapy.

### Potential efficacy in reducing fatigue in cancer survivors

Both the intention-to-treat (ITT) and per-protocol (PP) analyses showed a significant reduction in fatigue between T0 and T1 (i.e., mean differences of 10.4 and 9.7, respectively). Similar reductions were also found between T0 and T2 (i.e., mean differences of 9.4 and 9.8 based on ITT and PP analyses, respectively). About two-thirds of the cancer survivors showed a clinically relevant improvement in fatigue between T0 and T1 (i.e., ITT = 71.4% and PP = 60.0%) and between T0 and T2 (i.e., ITT = 57.1% and PP = 66.7%), which is well above the 45% that was set as a benchmark value. The means and SEs at all assessments and test parameters for ITT, PP, and sensitivity analyses can be found in Table 3.

**Table 3:** Intervention efficacy in reducing fatigue in cancer survivors

Outcome measures	Fatigue severity			Clinically relevant improvement (yes)	
	Mean (SE)	***T***	*p*^a^	***N***	**%**
*Intention to treat analyses (N* = *21)*					
T0	43.6 (1.8)				
T1	33.1 (1.8)				
T2	34.2 (1.4)				
T0−T1	10.4 (2.1)	5.007	< 0.001	15	71.4
T0−T2	9.4 (1.9)	4.959	< 0.001	12	57.1
*Per-protocol analyses (N* = *15)*					
T0	44.2 (2.2)				
T1	34.5 (2.2)				
T2	34.4 (1.9)				
T0−T1	9.7 (2.7)	3.614	< 0.001	9	60.0
T0−T2	9.8 (2.3)	4.169	< 0.001	10	66.7
*Sensitivity analyses*					
T0 (*N* = *17)*	43.7 (2.1)				
T1 *(N* = *17)*	33.3 (2.2)				
T2 *(N* = *13)*	34.7 (2.2)				
T0−T1	10.4 (2.5)	4.191	< 0.001	11	64.7
T0−T2	8.7 (2.6)	3.296	< 0.006	7	54.8

Intention-to-treat and per-protocol analyses are performed with imputed data. Sensitivity analyses represent analyses with unimputed data with those individuals with complete data at T1 and/or T2. Effect sizes for the sensitivity analyses are Cohen’s *d*_T0−T1_ = 1.0, Cohen’s *d*_T0−T2_ = 0.9

^a^For pooled data, only two-sided *p*-values are provided. For sensitivity analyses, one-sided *p*-values are < 0.001 and 0.003 for T0−T1 and T0−T2, respectively

### Feasibility of the trial procedures

Of the 21 couples who entered the study, 20 cancer survivors completed the baseline, 17 completed T1, and 13 completed T2. The fatigue questionnaire was completed by 81% (17/21) of the cancer survivors at T1 and 62% (13/21) at T2, which is above the benchmark values of 65% and 60%, respectively. The completion rate of the satisfaction with the intervention items was similar (i.e., 81% for cancer survivors). For partners, the completion rates for satisfaction with the intervention were lower (i.e., 52%; 11/21), but still above the critical value of 33%.

### Potential efficacy in promoting well-being in cancer survivors and partners

Explorative analyses also showed improvements in emotional well-being in cancer survivors and their partners. More specifically, there were statistically significant decreases in anxiety and depressive symptoms between T0 and T1 (*ds* = 0.65 and 0.44, respectively) in cancer survivors. Similar decreases in anxiety and depressive symptoms were found in partners (i.e., significant for anxiety from T0 to T2, and for depressive symptoms from T0 to T1 and T0 to T2). Relationship satisfaction did not change significantly over time. See Table 4 for more details.

**Table 4:** Means and SD emotional and relational well-being of cancer survivors and partners and statistics for changes over time

**Outcome measures**	**T0**	**T1**	**T2**	**T0−T1**		**T0−T2**	
	Mean (SD)	Mean (SD)	Mean (SD)	T (*p*)	***d***	T (*p*)	***d***
*Cancer survivors*	*n* = 20	*n* = 17	*n* = 13				
Anxiety	8.7 (3.4)	6.3 (3.8)	7.9 (3.4)	2.678 (0.008)	0.65	1.199 (0.127)	0.33
Dep Symp	7.1 (3.7)	5.2 (3.5)	5.5 (3.9)	1.832 (0.043)	0.44	1.775 (0.051)	0.49
Rel Satis	65.3 (9.6)	64.4 (13.3)	65.5 (9.4)	−0.338 (0.370)	−0.08	−0.337 (0.371)	−0.09
*Partners*	*n* = 19/18	*n* = 11	*n* = 13				
Anxiety	6.8 (4.1)	7.1 (2.6)	4.2 (2.8)	0.934 (0.186)	0.28	3.237 (0.004)	0.90
Dep Symp	6.2 (4.8)	5.6 (3.3)	4.1 (3.0)	2.046 (0.034)	0.62	2.341 (0.019)	0.65
Rel Satis	66.1 (8.6)	62.1 (12.3)	64.5 (10.7)	−0.254 (0.402)	−0.08	0.043 (0.483)	0.01

Unimputed data

*Dep Symp*, depressive symptoms; *Rel Satis*, relationship satisfaction

## Discussion

This single-arm pilot study examining the feasibility of the couples therapy COMPANION showed that 71% of the couples completed the therapy with the majority of the cancer survivors and partners being satisfied with the therapy and the couples’ approach. All partners showed at least the minimal required degree of involvement, and the majority of the partners (75%) showed high involvement. Furthermore, fatigue significantly reduced, with clinically relevant improvement in about two-thirds of the cancer survivors. Also, well-being in both cancer survivors and partners improved in terms of less anxiety and fewer depressive symptoms. Most couples were already highly satisfied with their relationship at the entry of the study, which did not significantly change over time, though the majority did indicate that doing the treatment together had a positive effect on their relationship. All variables to assess the acceptability of the intervention, potential efficacy, and feasibility of the trial procedures were above the benchmark values. One exception is the follow-up questionnaire completion rate of the partners, although it was above the critical value. The reason for this is not clear, but perhaps partners found the combination of weekly assessments plus follow-up questionnaires too intensive or felt it was redundant.

There are several reasons why couples eMBCT may show greater benefits for partnered cancer survivors than an individual eMBCT. The main reason would be that participating in therapy as a couple may increase the feeling of being a team that deals with the fatigue and its consequences together. The couple may reach a mutual understanding of what could be helpful in dealing with the fatigue, in terms of what the cancer survivor could do, what the partner could do, and what they could do as a couple. In line with this, our previous study indicated that cancer survivors felt that a couple approach would empower the partner to support the cancer survivor in coping with the fatigue and completing the therapy [23]. In that study, several cancer survivors reported that, particularly during the initial stages of their individual eMBCT, they struggled to find the words to express their experiences of fatigue and sharing them with their partner, as they were still deeply engaged in their own process of understanding and acceptance. Partners also reported a need for professional support in understanding the cancer survivor’s fatigue. By participating in therapy together, partners can be informed, cancer survivors can receive the necessary partner support, and couples can work better together. The current findings indicate that cancer survivors as well as their partners felt that they better understood the fatigue and had learned together how to deal with it in their daily lives (Appendix A, Table 1). Nevertheless, follow-up research on the working mechanisms of the efficacy of a couples’ approach is needed to investigate this explanation further.

It is likely that the couples’ approach does not only have benefits with respect to CCRF, but also improves the well-being of both partners and their relationship quality. In line with this, a recent meta-analysis examining couple interventions focusing on coping with cancer in general found that interventions that included couple communication, support, and skills building (cf. mindfulness exercises) components had a positive effect on reducing cancer-related distress [37]. In the current study, relationship satisfaction was already quite high at baseline. In other words, there may have been selection bias as couples with a good relationship were more likely to be interested in the couples’ approach [38]. Therefore, it may be worthwhile to put specific effort into recruiting couples that experience challenges in their relationship in which dealing with CCRF plays a role: perhaps, these couples would benefit even more.

Another beneficial element of partner involvement is that it may help keep the cancer survivor engaged in therapy and reduce dropout rates. Although we cannot exclude that the opposite might also happen, our results do indicate that the involvement of the partner may have served such a function, as at least half of the couples felt that the partners encouraged the cancer survivor to continue treatment. This could be important as previous studies investigating individual eHealth mindfulness-based programs (eMBP) showed considerable dropout up to 48% [39], and the average dropout rate has been found to be higher in eMBCT as compared to face-to-face MBCT [40]. However, online interventions do offer the flexibility [41] that the couples in the current study and our previous work [23] found important. In further research, it remains important to pay attention to the degree of flexibility. Too much flexibility may undermine the benefits of a truly dyadic approach, either by not involving the partner sufficiently or by placing too much emphasis on the partner and their problems.

Our study has a number of limitations that need to be dealt with in future research. As this is a pilot study, the sample of couples is small. Additionally, the absence of a control group limits the ability to determine changes in fatigue and well-being that may occur without therapy. These limitations restrict the conclusions that can be drawn regarding the effectiveness of COMPANION. More specifically, our results give us an indication of the efficacy of reducing fatigue, but this, as well as our suggestions about lower dropout in couples eMBCT as compared to individual eMBCT, needs further testing. In such a larger randomized controlled trial (RCT), differences in diseases and stages should also be taken into account. Owing to the small sample size, we also had to deviate from the protocol with respect to analyses of the weekly assessments. Unfortunately, analysis of these weekly assessments to investigate potential working mechanisms of couples eMBCT was not possible, but the data will be helpful in estimating the sample size needed for such analyses in a larger RCT.

In conclusion, our study results support the acceptability, feasibility, and potential efficacy of COMPANION. The added value of COMPANION versus the individual eMBCT focused on reducing CCRF in cancer survivors needs to be studied further in a randomized controlled trial with a larger representative sample. In case this future study supports our current findings, the intervention may be offered more broadly and complement much-needed psychosocial interventions to help couples cope with fatigue after cancer.

## Supplementary Information

Below is the link to the electronic supplementary material.

ESM 1 (16.2 KB DOCX)

## Data Availability

The data sets generated and analysed during this study are available from the corresponding author on reasonable request.
