# Supplementary material for: Adopting a couples’ approach to treating chronic cancer-related fatigue: a single-arm pilot trial of a web-based cognitive therapy for couples
Source: Support Care Cancer. 2026 Mar 16;34(4):328. doi: 10.1007/s00520-026-10547-8 (PMC12992381; doi:10.1007/s00520-026-10547-8)
Supplement: Supplementary file 1 — (16.2 KB DOCX) [file 520_2026_10547_MOESM1_ESM.docx]

**Supplementary Material**

Appendix A

Table 1. The Involvement of the Partner in the Therapy

| Aspects of involvement | Partner rating | Therapist rating |
| --- | --- | --- |
| The partner … (Yes/No) | N/% | % |
| 1. … encouraged the cancer survivor to start and/or continue the session | 6/55 | 47 |
| 1. … reminded the cancer survivor to do exercises or to keep appointments with the therapist | 5/45 | 27 |
| 1. … took over (household) tasks from the cancer survivor, so that he/she had time to do the treatment | 5/45 | 40 |
| 1. … read information from the treatment | 10/91 | 93 |
| 1. … discussed the content of the treatment with the cancer survivor | 8/73 | 73 |
| 1. … did exercises together with the cancer survivor | 7/64 | 67 |
| 1. … did exercises | 9/82 | 47 |
| 1. … did the joint session on communication (‘Fatigue and the other’) | 6/55 | 86 |
| 1. … has been in contact with the therapist together with the cancer survivor | 11/100 | 100 |
| 1. … has supported the cancer survivor in putting what he/she has learned into practice (e.g., how to set boundaries or to be active regularly) | 4/36 | 73 |

Note. In total, 11 partners filled out this questionnaire

Appendix B.

Table 2. Satisfaction of Participants with Different Aspects of COMPANION

| Aspects of COMPANION | Cancer survivor | Partner |
| --- | --- | --- |
|  | N (%) Satisfied | N (%) Satisfied |
| 1. The texts of the treatment were easy to understand | 16 (94) | 9 (82) |
| 1. The time investment was acceptable | 11 (65) | 7 (64) |
| 1. I would have liked it if my partner had had his/her own account /I would have liked it if I had had my own account | 7 (41) | 5 (45) |
| 1. The treatment has helped me to cope better with my fatigue /The treatment has helped my partner to cope better with his/her fatigue | 12 (71) | 6 (55) |
| 1. The treatment has helped me to cope better with the cancer and my partner’s fatigue | - | 6 (55) |
| 1. I think the treatment helped more because … my partner/I was involved | 8 (47) | 7 (64) |
| 1. I would have preferred to do the treatment all by myself /I think my partner would have preferred to do the treatment all by him/herself | 13 (76) | 9 (82) |
| 1. My partner liked that I was involved in the treatment | - | 9 (82) |
| 1. I enjoyed doing the exercises with my partner | 8 (47) | 7 (64) |
| 1. I liked that I could decide for myself in which parts … I wanted to involve my partner/I wanted to be involved | 10 (59) | 9 (82) |
| 1. Doing the treatment together had a positive effect on our relationship | 11 (65) | 6 (55) |
| 1. I would recommend the treatment to couples who are dealing with cancer-related fatigue | 13 (76) | 5 (45) |
| 1. My partner now understands/I now understand … better what it is like to be fatigued | 11 (65) | 7 (64) |
| 1. I have received tips on how to deal with my partner’s fatigue | - | 5 (45) |
| 1. My partner learned how to deal with my fatigue /I learned how to deal with my partner’s fatigue | 10 (59) | 5 (45) |
| 1. My partner helped me to gain insight into my fatigue /I was able to help my partner to gain insight into his/her fatigue (e.g., by talking about it) | 10 (59) | 5 (45) |
| 1. My partner encouraged me/I was able to encourage my partner … to start or continue treatment | 9 (53) | 5 (45) |
| 1. My partner reminded me of appointments or exercises /I reminded my partner of appointments or exercises | 5 (29) | 6 (55) |
| 1. My partner helped me/I was able to help my partner … to put what was learned into practice (e.g., how to maintain a fixed daily structure) | 3 (18) | 5 (45) |
| 1. My partner and I have learned together how to deal with fatigue in our daily lives (e.g., by dividing tasks) | 8 (47) | 5 (45) |
| 1. My partner received attention for problems that he/she was facing as result of my cancer and fatigue /I have received attention for problems that I myself am facing as result of my partner’s cancer and fatigue | 11 (65) | 7 (64) |
| 1. My partner and I have been able to pay attention to what the cancer and the fatigue are doing to our relationship | 12 (71) | 7 (64) |
| 1. It was difficult for my partner and me to make time for treatment together | 5 (29) | 2 (18) |
| 1. It was difficult to follow the treatment together because we differ too much in what we can and want to do (e.g., being able to perform exercises, being able to use the computer) | 9 (53) | 6 (55) |
| 1. Going through treatment together has created tensions in our relationship | 16 (94) | 9 (82) |
| 1. I felt like I was burdening my partner with the treatment /By participating in the treatment, I felt like I was burdening my partner with my own problems | 9 (53) | 10 (91) |
| 1. Because my partner was involved, I felt that I could not discuss everything openly with our therapist | 14 (82) | 10 (91) |
| 1. (Because my partner was involved,) there was too little attention for what I wanted to work on | 14 (82) | 9 (82) |

Note. N (%) Satisfied = the number (percentage) of 17 cancer survivors/ 11 partners with a score of 5 or higher on a positive item or a 3 or lower on a negative item, on a scale of 1 (completely disagree) – 7 (completely agree).
